# Prevalence of papular urticaria caused by flea bites and associated factors in children 1–6 years of age in Bogotá, D.C.

**DOI:** 10.1186/s40413-017-0167-y

**Published:** 2017-11-07

**Authors:** Evelyne Halpert, Elizabeth Borrero, Milciades Ibañez-Pinilla, Pablo Chaparro, Jorge Molina, Maritza Torres, Elizabeth García

**Affiliations:** 1 Dermatology Section, Hospital Universitario Fundación Santa Fe de Bogotá, Bogotá, Colombia; 2 Eje de Salud Pública, Fundación Santa Fe de Bogotá, Bogotá, Colombia; 3 Bioestadística y Epidemiología, CICS Universidad del Rosario, Bogotá, Colombia; 4 Escuela de Salud Pública, Maestría en Epidemiología, Universidad del Valle, Bogotá, Colombia; 5 Centro de Investigaciones en Microbiología y Parasitología Tropical, Departamento de Ciencias Biológicas, Universidad de los Andes, Bogotá, Colombia; 6 Centro de Investigaciones en Microbiología y Parasitología Tropical, Departamento de Ciencias Biológicas, Universidad de los Andes, Bogotá, Colombia; 7 Allergy Section, Hospital Universitario Fundación Santa Fe de Bogotá, Faculty of Medicine, Universidad de los Andes, Bogotá, Colombia; 8 Sección de Alergia Pediátrica, Fundación Santa Fe de Bogotá, Av 9 N° 116–20, oficina 213, Bogotá, D.C Colombia; 9 Universidad Manuela Beltrán, Bogotá, Colombia

**Keywords:** Papular urticaria, Flea allergy, Prevalence, Risk factors, Bogotá D.C

## Abstract

**Background:**

Papular urticaria is a chronic inflammatory disease caused by exposure to arthropod bites. The disease has been reported in children attending medical centers, but the causes as the risk factors associated with the disease have not been established. The objective of this study was to determine the prevalence of papular urticaria caused by flea bite and identify the risk factors in children between 1 to 6 years of age in Bogotá D.C, between March 2009 and June 2011.

**Methods:**

A cross-sectional, two-stage, clustered study using random probability sampling and stratified with proportional allocation was carried out in children (1–6 years of age) in educational institutions in Bogotá D.C. to determine the prevalence of the disease. Children underwent a dermatological examination by general practitioners with a previous training. Furthermore, digital photographs of skin lesions were taken for further confirmation of the diagnosis by dermatologists. A structured survey was completed by the parents or caregivers, and it was evaluated using an unconditional logistic regression to identify factors associated with the disease.

**Results:**

A total of 2437 children were included in the study. The prevalence of papular urticaria caused by flea bite in this population was 20.3% (CI 95%: 18.2 to 22.5%). The major risk factors associated with the disease were the presence of fleas in households (OR 1.74, CI 95%: 1.35 to 2.25), using mattresses without springs (OR 1.73, CI 95%: 1.20 to 2.50), the use of daily public transportation to carry the children to the educational institutions (OR 1.76, CI 95%: 1.07 to 2.89), having a soil/earth floor in the main bedroom (OR 6.81, CI 95%:1.16–39.96), and having siblings with a history of atopic dermatitis (OR 1.76 CI 95%: 1.07–2.89).

**Conclusions:**

A high prevalence of papular urticaria caused by flea bite was found in Bogotá D.C. The main factors associated with the disease might be modified with the implementation of prevention, control strategies in housing, educational institutions, and public transportation.

## Background

Papular urticaria is a chronic inflammatory disease caused mainly by exposure to the bite of arthropods [1]. Fleas and mosquito are the insects most commonly associated with this disease, but other insects also could cause the same skin reaction [1]. Papular urticaria is usually manifested during the first years of life, presenting an improvement normally at age of 7 years [1]. However, some case persists even into adulthood [2]. The disease is characterized by a hypersensitivity reaction that can be manifested most commonly through papule-type skin lesions, and sometimes through wheals, vesicles, blisters or scabs. Occasionally, as a result of the constant bites, the patient might develop hypo-or hyperchromic residual pigmentations in the skin with an intense pruritus, severe infections and scarring [2]. Currently, research advances on the immune pathogenesis of papular urticaria have been directed toward the study of the immune response, trying to identify a specific immunotherapy [3, 4].

Papular urticaria in children occurs mainly in tropical region, frequencies of 2.4 to 16.3, 4.4 and ≈5.2% have been observed in in Mexico city [2, 5], Mali [6], and India [7, 8], respectively. In Nigerian was reported a frequency of 14.6% in children with papular urticaria attending a dermatology clinic during 2008 [9]. Also, countries such as Pakistan, Thailand, and Tanzania has described frequencies of 1.99%, 2.3%, and 5.6%, respectively [10–12]. Overall, these studies established the frequency of dermatologic diseases in a hospital setting instead of a population level. The prevalence of papular urticaria in school children has been reported in Egypt, a study that included a survey of 6162 children in 30 schools reported a prevalence of 4.4% [13]. Similar studies in Nigeria showed a prevalence of 3.3% in 2005 and 8.5% in 2009 [14, 15].

In Bogotá D.C., an exploratory study was carried out in 30 households of patients with papular urticaria, and the results showed that fleas were presented in 50% of the households, especially in those with dogs [16]. Fleas in pets were detected using a handheld vacuum in different rooms in the house. Also, it was researched in the patient’s bedroom and the place where the animals slept, with a particular focus on corners, grooves in the floors, furniture, mattresses, and carpets [16]. Several studies have discussed that papular urticaria is a frequent reason for consultation in regions where fleas are common (2–15). However, to our knowledge, there are no prevalence studies for the disease in Colombia and Latin America, as well, environmental or individual risk factors have not been elucidated. Therefore, the objective of this study was to determine the prevalence of papular urticaria caused by flea bite and to identify the risk factors in children between 1 to 6 years old in Bogotá D.C.

## Methods

A cross-sectional study was performed in order to determine the prevalence of flea bite papular urticaria (FBPU) and its associated factors in children (1–6 years of age) attending public and private educational institutions in Bogotá D.C., during March 2009 to June 2011. The sampling included educational institutions with a registration to the District Departmental of Education in 2009, as well as, nurseries and kindergartens registered with the Ministry of Social Integration and the Colombian Family Welfare Institute in Bogotá D.C. A multistage probability and a randomized sampling design in two stages were used. The strata were defined according to the neighborhood and type of institution (public or private) and two stages were designed. In the first, the primary sampling units were the educational institutions selected through a simple random sampling and weighted by the institution size within the strata. Bogotá D.C. is divided into 19 localities with neighborhoods classified in six socioeconomic strata according to urban characteristics such as population density, quality of public areas and housing characteristics (stratum one corresponds to the lowest and stratum six to the highest). In the second stage (stratification with proportional allocation), the secondary sampling units were children of 1 to 6 years old enrolled in the previously selected institutions, chosen by proportional allocation by locality and type of institution (public and private). Clusters were educational institutions, as well as, nurseries and kindergartens. A sample size of 2437 children was calculated taking into account a papular urticaria prevalence of 16.3% [2], an accuracy of 5%, relative standard error (e_r_) of 11.1%, statistical confidence of 95% and a design effect by clusters of 1.5.

To determine the prevalence of FBPU, patients were diagnosed using clinical parameters as follows: skin lesions such as hives, papules, papule-vesicles, pruritic and grouped blisters, presenting a chronic course, often leaving abrasions, scabs, hypo-or hyperchromic macules, and mainly located in the trunk and limbs. These papules were most often located in areas where clothing fits snugly, such as under the socks and the waistband. In some patients, exposed areas of the body extremities were also affected [7, 17]. As a case, it was considered a patient diagnosed with FBPU by physical examination. A control included a child without papular urticaria at the physical examination. Children with less than 1 year old of residence in Bogotá and with concomitant diseases such as malignancies, extensive active atopic dermatitis, and active systemic infections were excluded.

Initially, the educational institutions, kindergartens and day cares were contacted, a presentation about the study was done, and posteriorly a consent from the school directors was obtained. For the data collection, a group of interviewers was trained. An interview with the parents or caregivers was done previously to the signing of the informed consent forms. The survey included variables related to sociodemographic data (Table 1), domestic animals associated with housing household (dogs, cats, rabbits and others), rodents (rats or mice at home or around the house), public services (Aqueduct, sewerage and garbage collection), housing characteristics (flooring material: wood, carpet, tile, cement or soil/earth in the different parts of the house such as bedroom, living room, kitchen and others; house or apartment; numbers of bedroom, adjacent house and others), mattress material (springs), presence of fleas in housing, type of transportation and personal history or family history of asthma, allergic rhinitis, atopic dermatitis and papular urticaria.

**Table 1:** Sociodemographic characteristics of the pediatric population sampled in Bogotá D.C.

Sociodemographic Characteristics	Number (*n*)	Percentage (%)
Sex		
Male	1211	49.7
Female	1226	50.3
Age (years)		
1–2	218	8.9
3–4	775	31.8
5–6	1444	59.3
Socioeconomic stratum		
1–2	1280	52.5
3–4	1007	41.3
5–6	150	6.2
Membership of Social Security		
Yes	2271	93.2
No	165	6.8
Scheme affiliation		
Contributory^a^	1770	72.7
Subsidized^b^	411	16.9
Private^c^	3	0.1
None	162	6.6
Other^d^	89	3.7
Number of people living with the patient		
1 to 4	1895	77.8
5 or more	541	22.2

^a^ Contributory: Regime to provide health insurance coverage for formal sector workers and their dependents, autonomous workers with a steady source of income from not poor households

^b^ Subsidized: Regime to provide health insurance coverage of individuals from poor households without employment in the formal sector, indigenous people and other vulnerable groups

^c^ Private: It refers to the prepaid medical plans

^d^ Other: Includes the regime to provide health insurance coverage of workers and their dependents from public corporations and autonomous institutions (i.e., armed forces, public oil corporation, etc.)

The medical examination as well the clinical history were performed in the educational institution by doctors previously trained by dermatologists in the correct diagnose of papular urticaria or other dermatologic diseases. As a part of the physical exam, pictures of the skin lesions were taken and reviewed by a trained dermatologist to confirm the diagnosis.

### Statistical analysis

The statistical analysis considered the weight and used of the module of complex samples from the SPSS version 20. In order to describe the variables, measures of frequency, central tendency and dispersion were used. The prevalence of papular urticaria was calculated using a 95% confidence interval. The estimation accuracy for prevalence was evaluated using the relative standard error, taking as criteria the international standards of statistical research from Canada [18]. To assess the association between FBPU and the risk factors, a bivariate analysis was carried out by calculating odds ratios (OR) with 95% confidence intervals.

In the multivariate analysis, a multiple unconditional binary logistic regression was used in order to verify the risk factors associated with FBPU, adjusting by confounder factors, and the collinearity between the multiple risk and protective factors were evaluated with the changes in betas and OR of the model. The risk factors that entered the model were the variables that showed a greater association in the bivariate analysis, defined by a *p* value <0.20, and the final model was selected using hierarchical modeling. Statistical tests were evaluated with a significance level of 5% (*p* < 0.05).

The present study meet the terms of the ethical requirements in accordance with Law 8430 of 1993 established by Colombian legislation. Additionally, it was approved by the Ethics Committee from the Fundación Santa Fe de Bogotá.

## Results

A total of 2437 surveys were taken among the parents or caregivers of children in the 19 localities of Bogotá D.C., having a slightly higher distribution in private educational institutions in contrast with public educational institutions (55.2% vs 44.8%, respectively). The mean age of the children was 4.49 ± 1.33 years (median = 5 years old). The sociodemographic characteristics of the 2437 children studied are shown in Table 1. Households had characteristics as tile was the predominant flooring in living and dining rooms, master bedrooms and children’s bedrooms (62.3%, 53.0%, and 52.8% respectively). In 32.1% of the households, the presence of at least one pet was confirmed (18.8% had dogs and 6.1% had cats). The presence of peri-domiciliary animals and having observed rodents in the accommodation were manifested in 20.5% and 18.8%, respectively. Almost all households had satisfactory public services, 99.1% presented a water supply via aqueduct, 98.7% had a sewerage service and 99.5% reported a garbage collection service. The clinical examination of the children showed that 42.7% of them had skin lesions. Of the total group studied, FBPU was diagnosed in 20.3% (95% CI: 18.2–22.5%). A family history of allergic rhinitis was found in 35.9%. A family history of papular urticaria was registered in 13.6%, and 14.3% of children had a previous diagnosis of atopic dermatitis.

The structured survey showed that in 35.7% of the households the presence of fleas was reported, and also that 43.5% of the family members living with the children reported having been regularly bitten by fleas. In addition, 50.7% of the parents or caregivers of the child traveled daily by bus, and 12.3% used private transportation. Risk factors included a low socioeconomic stratum, soil/earth floor, the presence of fleas in the household, family members with flea bite, siblings with atopic dermatitis and springless mattresses (Table 2). Protective factors were: wooden floors, the presence of sewerage, no presence of neighboring houses, and private transportation (Table 3). A multivariate analysis showed that risk factors associated with FBPU were the detection of the presence of fleas in the accommodation (OR 1.72, 95% CI 1.32 2.24), use of mattresses without springs (OR 1.73, 95% CI 1.19–2.50), daily use of public transportation (OR 1.76, 95% CI 1.07–2.92), having a soil /earth floor in the main bedroom (OR 6.61, 95% CI 1.14–38.50), and siblings with a history of atopic dermatitis (OR 1.46, 95% CI 1.01–2.11) (Table 4).

**Table 2:** Risk factors associated with papular urticaria in children (1–6 years of age)

Factors	Prevalence	CI 95% for Prevalence	Relative standard error (%)	*p* value	OR	CI 95% OR
Socioeconomic stratum						
1 to 3	21.1	18.9–23.6	5.7	0.004	1.75	1.20–2.56
4 to 6	13.3	9.7–17.9	15.3		1.00	
Soil/earth floor in the living room						
Yes	70.5	24.3–94.7	29.8	0.009	9.35	1.23–71.43
No	20.3	18.1–22.7	5.6		1.00	
Soil/earth floor in the main bedroom ^a^						
Yes	70.1	28.5–93.2	26.6	0.003	9.35	1.55–92.59
No	20.1	18.0–22.4	5.4		1.00	
Mattress material: Without springs ^b^						
Yes	24.1	20.0–28.8	9.2	0.025	1.48	1.05–2.10
No	17.6	14.9–20.7	8.2		1.00	
Members of the family usually bitten by fleas						
Yes	23.0	19.6–26.7	7.8	0.030	1.34	1.02–1.74
No	18.2	15.8–20.9	7,0		1.00	
Presence of fleas in households						
Yes	26.9	23.6–30.4	6.4	< 0.001	1.84	1.42–2.38
No	16.6	14.1–19.5	8.1		1.00	
Siblings with atopic dermatitis						
Yes	27.2	21.1–34.3	12.2	0.019	1.51	1.07–2.13
No	19.9	17.7–22.2	5.6		1.00	

^a^Soil Floor (earth floor)

^b^Without springs (foam, speck [mattresses made with leftover pieces of material such as cloth, wool, specks, etc.)

**Table 3:** Protective factors against papular urticaria in children (1–6 years of age)

Factors	Prevalence	CI 95% for Prevalence	Relative standard error (%)	*p* value	OR	CI 95% OR
Presence of rodents in the bedroom						
Yes	8.6	3.9–17.9	38.7	0.02	0.36	0.15–0.87
No	20.5	18.2–22.9	5.7		1.00	
Sewerage system						
Yes	20.1	18.0–22.3	5.4	0.023	0.43	0.20–0.90
No	36.6	21.9–54.2	22.9		1.00	
Wooden floor in the living room						
Yes	13.5	9.1–19.4	19.1	0.016	0.56	0.35–0.90
No	21.6	19.2–24.1	5.7		1.00	
Wooden floor in the main bedroom						
Yes	15.3	11.8–19.6	12.8	0.016	0.66	0.48–0.92
No	21.3	19.0–23.8	5.7		1.00	
Wooden floor in the children’s bedroom						
Yes	14.2	10.7–18.8	14.2	0.004	0.59	0.41–0.84
No	22	19.4–24.7	6		1.00	
Adjacent house						
Without	15.3	11.9–19.5	12.5	0.006	0.62	0.44–0.87
With	22.5	20.0–25.3	5.9		1.00	
Private transportation used to travel on a daily basis						
Yes	15.2	11.5–19.8	13.8	0.012	0.63	0.448–0.904
No	22.0	19.6–24.6	5.7		1.00	
Transportation by bus owned by the educational institution, used on a daily basis by children						
Yes	11.4	7.7–16.6	19.5	0.001	0.47	0.29–0.75
No	21.3	19.1–23.8	5.6		1.00	
Private transportation used by children to go to the educational institution						
Yes	12.3	7.8–18.8	22	0.010	0.51	0.31–0.85
No	21.4	19.2–23.8	5.4		1.00

**Table 4:** Multivariate model for papular urticaria in children (1–6 years of age) in Bogotá D.C.

Variables	*B*	Standard Error	*p* value	OR	CI 95%
Fleas in housing					
Yes	0.542	0.132	< 0.001	1.72	1.32–2.24
No				1.00	
Soil/earth floor in the main bedroom					
Yes	1.889	0.886	0.036	6.61	1.14–38.50
No				1.00	
Mattress materials					
Without springs	0.546	0.186	0.004	1.73	1.19–2.50
With springs				1.00	
Transportation					
Public	0.567	0.253	0.028	1.76	1.07–2.92
Private				1.00	
Siblings with atopic dermatitis					
Yes	0.378	0.187	0.046	1.46	1.01–2.11
No				1.00	

## Discussion

In the present study, a prevalence of papular urticaria of 20.3% was observed in children between 1 and 6 years of age. This prevalence is high in comparison to the prevalence reported in previous studies, and it could be due to several of those reports included children with a higher average age [13–15], and it has been reported a reduction in the immunological reaction to insect bite after the seventh year of life [2, 19].

Even though there are few published studies about the risk factors associated with papular urticaria, having pets at home has been proved a predisposing factor for insect bite dermatitis; another risk factor has been residing in an area of heavy insect infestation, warm weather, spring and the use of perfumes and colognes [20]. In contrast, 32.1% of the households confirmed the presence of at least one pet, but, pets at home were not a risk factor. In general, pets and rodents have been also considered a risk factor [21], especially on the *Rattus norvegicus, Rattus rattus, Mus musculus*, dogs, and cats have been collected species of *Pulex irritans, Ctenocephalides catis, Ct. Cannis and Xenopsylla cheopis*; highlighting the possibility the interchange of fleas in the hosts around the houses. However, surprisingly in our study, the rodents behaved as a protective factor in the in the bivariate analysis and it was discarded from the multivariate analysis. To our knowledge, this is the first study with the objective of identifying risk factors for papular urticaria caused by flea bite. We found that having a soil /earth floor in the main bedroom is a risk factor for the disease and it has been reported that dirty floors are a risk factor associated with FBPU due to the close association between these conditions and the presence of immature fleas feeding and continuing its metamorphosis under favorable environmental parameters [22].

A history of atopic dermatitis in siblings was found as other risk factors for FBPU; however, in the tropics, it is difficult to clearly diagnose atopic dermatitis [23], and also, it could be limited by recall as well a diagnostic bias. Others risk factors were the use of public transport for transportation of the children, the use of mattresses without springs or foam lining, and the detection of fleas inside the accommodation (Table 2). Among the risk factors, we would highlight the presence of fleas in view of the fact that patients attending the Pediatric Dermatology and Allergy division of the Fundación Santa Fe de Bogotá D.C. have showed papular urticaria immune and histopathological reactions when exposed to flea antigens [3, 16, 19]. These results support that fleas can be involved in those reactions of papular urticaria frequently diagnosed in this dermatological practice all year round.

Mosquitoes and bedbugs are other insects associated worldwide with papular urticaria [1], but only *Culex quinquefasciatus* has been reported in Bogotá D.C. [24]. The differentiation between mosquitoes and fleas bite is not totally accurate but just suggested by clinical identification of corporal distribution of the bites. However, in our study, we were able to discriminate between the skin reactions caused by bites insects and fleas, by assessing the clinical manifestations of the reactions, the presence of the papules in body areas where clothing fits snugly, such as the trunk and limbs. In only a few patients were exposed areas of the body extremities also affected. For flying insects such as mosquitoes, the usual sites of bite are the exposed areas of skin, with single lesions tending to be the majority of the cases [25]. On the contrary, the trunk may be involved in the case of bedbug bites and they may leave single or double bite marks [25]. Bedbug bites are usually multiple, painless and linear in configuration [1]. A row of three bedbug bites is sometimes referred to as the ‘breakfast, lunch, dinner’ sign [1, 26, 27]. Also a predilection for the eyelid has been reported in the case of bedbugs, perhaps because this area is the only one exposed when a child is sleeping [28]. Flea bite can sometimes also cause papules in groups of three [29], but in these reported cases the presence of papular urticaria due to flea bite was associated with the presence of pets [29–31]. As a limitation in the present study, information about the presence of mosquitoes or bedbugs inside the houses was not asked. Even though, household fleas and papular urticaria associated with their bites have been reported previously [29–32], this study reports for the first time the relationship between the presence of fleas in households and the clinical diagnosis of the disease demonstrated in Bogotá D.C. Fleas are highly adaptable to different environmental domiciliary conditions and a favorable weather conditions in Bogotá D.C., could make possible flea reproduction and growth in this region [33], as has been previously shown [16].

Our results also showed an association between public transportation and FBPU in Bogotá D.C. Different studies have reported the presence of fleas and other insects on board ships and aircraft [34, 35]. No literature has been published on fleas in public transportation in Bogotá D.C.; however, the presence of fleas in buses and other services has been well known. The continual flow of people in the available infrastructure will certainly favor the spread of fleas [36–38]. The presence of fleas associated with the type of flooring material (especially soil /earth floor) is consistent with difficulty maintaining hygienic conditions [38, 39]. In most cases, immature stages of fleas are found entirely off-host and feeding on organic matter that can be found on the soil floor of the burrow and/or the nest of the host [40]. These conditions provide immatures with the environmental conditions necessary for their postembryonic development [40]. In this study, housing with soil floors was associated with FBPU in Bogotá D.C., although the CI of 95% was wide because households presenting this characteristic were not very frequent. The negative phototactic responses as a positive geotropism characteristic of the larvae may help to explain the association between fleas and the material of mattresses. Protective and environmental conditions sought by the larvae can be found in the deepest part of rugs, mattresses and stuffed furniture [41, 42]. In 2005, Bogotá D.C. had 9.2% of the population with unsatisfied basic needs [43], and 24.8% of the population lived in conditions below the poverty line; besides, the city showed great contrasts between economic strata, ethnic-cultural groups and geographical areas [44]. Housing occupied by the poorest populations possesses dirt floors, indicating that poverty is strong enough to be selected as a risk factor associated with FBPU, as has been shown by Naafs 2006 in the case of urticaria papular associated with insect bites in the tropics [22]. Something similar is observed in Colombia between lack of sewerage and poverty [45, 46].

Atopic dermatitis in siblings was another factor associated with FBPU in our study (Table 2). Family history has always been considered an important risk factor for the development of allergic diseases such as asthma, allergic rhinitis and atopic dermatitis [47]. However, this study found no association between the presence of asthma and allergic rhinitis in children and their families with FBPU. As in other studies, atopic dermatitis may or may not be associated with asthma and/or allergic rhinitis [48]. On the other hand, other related arthropods with urticaria papular has been associated with allergic diseases such as mosquitoes and caddisflies [49]. Future studies might focus on the relationship between atopic dermatitis and FBPU. In accordance with the bivariate analysis results, the occurrence of the highest prevalence of papular urticaria in socioeconomic strata (one to three) in children living in households with soil floors in the main areas, and in those households had presence of fleas suggest a possible relationship of FBPU with adverse socioeconomic conditions. Multivariate analysis confirmed the strong statistical significance of the presence of fleas in housing and the mattress material, and also there was a high OR for soil floor in the main bedroom, although the CI was very wide (Table 4).

Together, these findings raise the possibility of a strong association between socioeconomic status in Bogotá D.C. and FBPU (Table 2). To confirm this association a rigorous socioeconomic stratification and housing typology should be prioritized in future studies. Although, in the field of dermatoses in Colombia, this is the first population-based, cross-sectional study carried out in this age group, some limitations of the study can be highlighted. For instance, it was not possible to establish a change over time for the risk factors, nor to include children in this age group who are not attending educational institutions. Finally, in our study, the protective factors for FBUP were Sewerage system, wooden floor, adjacent house and private transportation. It was found that the protective factors for insect bite dermatitis are the use of full sleeve clothes and keeping the doors and windows closed at night [43].

It is important to implement strategies in order to prevent and control the presence of fleas in housing, educational institutions and public transportation, as recommended by the World Health Organization [39]. Papular urticaria is a preventable disease and it is strongly recommended that school teachers, school nurses and family members are trained to recognize the symptoms of the disease, to carry out cleaning activities and to ask for control campaigns against fleas in schools and homes. At the level of policy makers, it is highly recommended to include in public health policies the control of fleas in public schools and public transportation in order to prevent the exposure of the children to fleas. In addition, all efforts carried out to reduce the level of poverty in the city and improve in living conditions of the citizens (sewerage and appropriate social housing) will be conducive to a decrease in the prevalence of papular urticaria in Bogotá D.C. as it has been reported in other countries [5, 14, 50]. An increase in skin diseases as a consequence of the deterioration in socioeconomic conditions has also been reported [51]. A reduction of fleas in Bogotá D.C. could have an effect on the prevalence of FBPU, as well as on the costs imposed on the health system as a consequence of the repeated treatments needed, as well as indirect costs caused by the absence from work of parents or caregivers.

## Conclusions

In this study a high prevalence (20.3%) of papular urticaria in children (1–6 years of age) in Bogotá D.C. was shown. The high prevalence of papular urticaria was associated with risk factors, such as the presence of fleas in housing, the use of public transportation, the presence of soil/earth floors, the use of foam mattresses and the report of atopic dermatitis in siblings. Papular urticaria is a preventable disease and it is highly recommended to implement strategies in order to prevent and control the presence of fleas in housing, educational institutions and public transportation in Bogotá D.C.

The cross-sectional nature of the study limits inferences, and a longitudinal design would have been better suited to test some associations.

## Abbreviations

Bogotá D.C.: Bogotá Distrito Capital
CI: confidence interval
FBPU: flea bite papular urticaria
OR: odds ratio
